# Pathogenetic Mechanisms Implicated in Sjögren’s Syndrome Lymphomagenesis: A Review of the Literature

**DOI:** 10.3390/jcm9123794

**Published:** 2020-11-24

**Authors:** Ioanna E. Stergiou, Aikaterini Poulaki, Michael Voulgarelis

**Affiliations:** Department of Pathophysiology, School of Medicine, National and Kapodistrian University of Athens, 11527 Athens, Greece; stergiouioanna@hotmail.com (I.E.S.); aikaterini.poulaki@gmail.com (A.P.)

**Keywords:** Sjögren’s Syndrome, lymphoma pathogenesis, mucosa associated lymphoid tissue, germinal centers, autoimmunity

## Abstract

Sjögren’s Syndrome (SS) is a chronic autoimmune disorder characterized by focal mononuclear cell infiltrates that surround the ducts of the exocrine glands, impairing the function of their secretory units. Compared to other autoimmune disorders, SS is associated with a notably high incidence of non-Hodgkin lymphoma (NHL) and more frequently mucosa associated lymphoid tissue (MALT) lymphoma, leading to increased morbidity and mortality rates. High risk features of lymphoma development include systemic extraepithelial manifestations, low serum levels of complement component C4 and mixed type II cryoglobulinemia. The discrimination between reactive and neoplastic lymphoepithelial lesion (LEL) is challenging, probably reflecting a continuum in the evolution from purely inflammatory lymphoid infiltration to the clonal neoplastic evolution. Early lesions display a predominance of activated T cells, while B cells prevail in severe histologic lesions. This strong B cell infiltration is not only a morphologic phenomenon, but it is also progressively associated with the presence of ectopic germinal centers (GCs). Ectopic formation of GCs in SS represents a complex process regulated by an array of cytokines, adhesion molecules and chemokines. Chronic antigenic stimulation is the major driver of specific B cell proliferation and increases the frequency of their transformation in the ectopic GCs and marginal zone (MZ) equivalents. B cells expressing cell surface rheumatoid factor (RF) are frequently detected in the salivary glands, suggesting that clonal expansion might arise from antigen selection of RF-expressing B cells. Abnormal stimulation and incomplete control mechanisms within ectopic lymphoid structures predispose RF MZ like cells to lymphoma development. Immunoglobulin recombination, somatic mutation and isotype switching during B cell development are events that may increase the translocation of oncogenes to immunoglobulin loci or tumor suppressor gene inactivation, leading to monoclonal B cell proliferation and lymphoma development. Concerning chronic antigenic stimulation, conclusive data is so far lacking. However immune complexes containing DNA or RNA are the most likely candidates. Whether additional molecular oncogenic events contribute to the malignant overgrowth remains to be proved.

## 1. Introduction

Sjögren’s Syndrome (SS) is benign autoimmune disease characterized by organ specific as well as systemic manifestations. Of interest this benign autoimmune disease can potentially lead to lymphomagenesis. A major complication in the progression of SS is lymphoma development, leading to increased mortality of SS patients that develop lymphoma in the course of their disease [1,2].

B cells displaying a marginal zone (MZ) phenotype are detected in the majority of lymphomas developing in the setting of SS. Mucosa associated lymphoid tissue (MALT) lymphomas account for 65% of SS related lymphomas, followed by diffuse large B cell lymphomas (DLBCL) and nodal MZ lymphomas [3,4]. Of note, most cases of DLBCL are probably the evolution of low-grade MZ lymphomas [5,6]. Since MALT lymphomas account for the majority of SS associated lymphomas, the pathophysiologic mechanisms described in this review mainly refer to this type of lymphoma.

Compared to patients with other autoimmune diseases, namely systemic lupus erythematosus (SLE) and rheumatoid arthritis (RA), patients with primary SS (pSS) present a higher risk for lymphoma development [7]. Several clinical and laboratory parameters have been proposed to distinguish patients at this high risk [3,8,9,10,11,12,13,14,15,16,17,18,19,20,21,22,23,24].

SS is characterized by lymphocytic infiltration of the exocrine glands. Lymphoepithelial sialadenitis (LESA), a notable histological feature of SS, is characterized by the presence of lymphoid populations surrounding and infiltrating the salivary ducts, along with disorganization and proliferation of the ductal epithelial cells [25]. The spectrum of histopathologic features of LESA ranges from a fully benign lymphoid infiltrate, sometimes associated with lymphoid follicular structures, that does not display immunoglobulin (Ig) light chain restriction in B-cells, to lymphoproliferative lesions with presence of centrocyte-like cells with or without areas of Ig light chain restriction in B cells [26]. B-cell clones are found in approximately 50% of LESAs without morphological or clinical evidence of lymphoma [26,27]. Monoclonal B cell expansion does not necessarily constitute lymphoma. The evolution from persistent polyclonal B cell activation to monoclonal B cell expansion and thereafter lymphoma development is a multistep process.

## 2. Predictors of Lymphoma Development

Several clinical and biological parameters can distinguish SS patients at higher risk for lymphoma development (Table 1).

The main clinical predictors include permanent parotid enlargement [3,9,10,11], splenomegaly [13], lymphadenopathy [3,9,10,11,12,13,21], palpable purpura [8,9,14], and peripheral neuropathy [15].

Biological predictors of lymphoma development include cryoglobulinemia [8,13,16,17,18,21,22], lymphopenia [14,16,17,19], low complement levels [8,9,11,13,14,15,17,19,21] and monoclonal component in serum or urine [11,15,16,17,20]. The presence of germinal center (GC)-like structures in minor salivary gland (MSG) biopsy and the Focus Score (FS) have also been recognized as possible predictors for lymphomagenesis in SS [23,24,28].

Disease activity assessed by the EULAR (European League Against Rheumatism) SS disease activity index (ESSDAI) was shown to be a predictor of lymphoma development [29].

The question relies on how do predictors of lymphoma development in SS correlate with pathophysiology of lymphomagenesis?

## 3. Pathogenetic Mechanisms Implicated in SS Lymphoma Development

### 3.1. The Role of Epithelial Cells

Lymphoepithelial lesions (LEL) comprise a salivary gland (SG) histological finding characteristic of SS, depicting the continuous interaction between epithelial and lymphoid cells. The discrimination between reactive and neoplastic LEL is challenging, probably reflecting a continuum in the evolution from purely inflammatory lymphoid infiltration to clonal neoplastic evolution. In this stepwise process, epithelial cells are not merely the target of the immune-mediated destruction of the SG secretory units, since evidence exists that the disorganized salivary epithelial cell in SS participates both in the induction of SG inflammation and the neoformation of lymphoid tissue [30]. While these cells have not been shown to function as antigen-presenting cells, they possess the capacity to do so [31]. For example, they express CD40 and adhesion molecules, and produce lymphoid chemokines, cytokines and B-cell activating factors, all of which indicate a potential role in the recruitment of dendritic cells (DCs), T and B cells in the inflamed glands, and the formation of ectopic lymphoid structures [30,32,33,34].

Elevated levels of Bcl-2 (B-cell lymphoma 2) associated X protein (BAX) in SS acinar endothelial cells drive them to apoptosis [35]. It has been demonstrated that SG epithelial cells constitutively secrete exosomes, thus presenting intracellular autoantigens [36]. Through apoptosis and formation of membrane-bound exosomes the salivary epithelium presents intracellular autoantigens, a process that prompts the break-down of immune tolerance, triggering the production of autoantibodies by infiltrating B cells [36].

Epithelial cells have an active role in recruiting lymphocytes that are present in SS SG lesions. Apart from expressing “lymphoid” chemokines that attract T cells, responsible for the initiation of the inflammatory process in SG, epithelial cells have been shown to express B cell attracting chemokine (BCA)-1 mRNA, a chemokine participating in lymphoid follicle formation [30]. Different patterns of chemokine expression could discriminate reactive from neoplastic lymphoid proliferation. Barone et al. demonstrated that expression of CXCL13 and CCL21 in the salivary glands of SS patients is indicative of reactive lymphoid aggregates and suggestive of their implication in ectopic lymphoid tissue organization, whilst CXCL12 expression predominates in infiltrated ducts and malignant B cells, possibly regulating the survival of malignant B cells [37]. A clear association of Fc receptor-like 4 positive (FcRL4^+)^ B cells and the epithelium has been demonstrated in patients with primary SS, with its importance being emphasized by its maintenance in parotid MALT lymphoma [38]. In SS the ductal epithelial cells express an array of chemokines, such as CCL3 and CCL5, that act as ligands for chemokine receptors, namely CCR1 and CCR5 respectively, expressed by FcRL4^+^ B cells [39,40]. FcRL4^+^ B cells are found in the peripheral blood of healthy individuals, making the above-mentioned chemokine-chemokine receptor interaction a potent mechanism contributing to homing and retention of FcRL4^+^ B cells to the ductal epithelium. Ductal epithelial cells of the affected SGs secrete high levels of CXCL10, the ligand of CXCR3 [41]. Circulating FcRL4^+^ B cells of pSS patients demonstrate expression of CXCR3, providing an explanation for the homing of these B cells to the ductal epithelial cells of the SGs [42].

Studies have shown that B-cell-activating factor (BAFF) expression by epithelial cells is induced directly by viral infection [43] or after interferon (IFN) stimulation [44] in SG of SS, suggesting that they participate in B cell activation. FcR4^+^ B cells, found in close proximity to the epithelium and expressing transmembrane activator and CAML (calcium-modulator and cyclophilin ligand) interactor (TACI), may be activated by the binding of BAFF to TACI, gaining a proliferation advantage [42]. Salivary gland epithelial cell (SGEC) lines from SS patients have also been shown to demonstrate up-regulated surface CD40 expression, thus participating in the induction and maintenance of the lymphocytic infiltrates [32].

Haacke et al. demonstrated that FcRL4^+^ B cells are in close association with the epithelium in the salivary gland tissue of pSS, an association maintained in pSS parotid MALT lymphomas, highlighting the importance of the epithelial-B cell interaction [38]. In SG MALT lymphomas, expansion of neoplastic cells takes place in LEL. The importance of the epithelium-B cell interaction is confirmed by the fact that FcRL4^+^ B cells of parotid MALT lymphomas maintain their association with the epithelium, since they are located within or in close proximity with the ductal epithelium that forms LELs [38].

The above-described mechanisms are pathophysiologic drivers for LEL development, as well as contributors to lymphomagenesis.

### 3.2. The Role of Dysregulated T Cells

The lymphocytic infiltrates of the MSGs in SS are mainly constituted of CD4^+^ T cells, which appear to be activated as they demonstrate increased expression of activation molecules, such as major histocompatibility complex (MHC) class II, cell adhesion molecules and cytokines [45,46]. CD40 ligand (CD40L) is expressed on activated T cells and it has been shown that T cells infiltrating the salivary glands of SS patients express CD40L, indicative of their activated state [47,48]. CD40 drives the activation, proliferation, and differentiation of B cells and rescues them from apoptosis [49]. In the setting of SS, increased B cell proliferation driven by the CD40/CD40L interaction may enhance the tendency towards lymphoma development. Lower Foxp3^+^ T-regulatory cell levels in MSG lesions of SS patients have been correlated with adverse predictors for lymphoma development, such as C4 hypocomplementemia and SG enlargement [50]. T follicular helper (Tfh) cells drive B cell selection and differentiation into memory and plasma cells in the process of antibody affinity maturation [51]. The frequency of sCD4^+^CXCR5^+^ Tfh in the SG of SS patients has been positively correlated with the presence of CD19^+^CD27^+^ memory B cells and CD19^+^CD27^high^ plasma cells, unraveling their possible implication in the formation of ectopic GCs [52].

### 3.3. B Cell Dysregulation

#### 3.3.1. Aberrant Distribution of B Cell Subsets

SS patients demonstrate aberrations in the distribution of B cell subsets, with CD27^+^ memory B cells being reduced in the circulation and increased in the SGs, while only SS patients with concurrent lymphoma show an increase in circulating CD27^+^ B cells [53]. This characteristic pattern of B cell distribution possibly reflects the accumulation of CD27^+^ memory B cells in the inflamed SG tissue. Bohnhorst et al. demonstrated that patients with SS are characterized by disturbances in the proportion of circulating B cell subpopulations, with high percentage of activated B cells, a phenomenon that might reflect a disturbance in B cell trafficking and/or alteration in B cell differentiation, possibly contributing to lymphoma development [54].

FcRL4^+^ B cells are more abundant in the parotid glands compared to labial SGs, providing a possible explanation of the preferential development of MALT lymphomas in the parotids [38]. The question relies on whether FcRL4 activation initiates at extra-glandular sites followed by their migration to the inflamed SS of pSS patients or whether it initially takes place locally in the SGs. Gene expression pathway analysis demonstrated that glandular FcRL4^+^ B cells of pSS patients had higher expression of genes involved in cell trafficking, B cell activation and Nuclear Factor kappa b (NFκ-B) pathway [42].

#### 3.3.2. Autoantibody Producing B Cells

MALT lymphomas develop at sites of chronic antigenic stimulation. It has been shown that the IgV heavy and IgV light chain genes of MALT lymphomas bear mutation patterns indicative of antigen (Ag)-based selection [55,56,57].

Ag driven activation and proliferation of B lymphocytes is one of the hallmarks of SS [58,59]. This process results to the production of autoantibodies, namely rheumatoid factor (RF), anti-Ro/SSA and anti-La/SSB, that are detected in the serum of SS patients [60]. B cell deregulation in SS patients is depicted by the presence of circulating immune complexes (IC), hypergammaglobulinemia, alterations in peripheral B cell subpopulations, oligoclonal B cell expansion and the well described increased risk of B cell non Hodgkin Lymphoma development [7,53,54,58,61,62,63].

SS patients display monoclonal immunoglobulins or light chains in their sera from early disease, as well as monoclonal mixed cryoglobulins with the presence of IgMk RF, an autoantibody that recognizes epitopes in the Fc region of IgG [64,65]. This suggests both monoclonal and polyclonal B cell activation early in the disease course. Hence, B lymphocytes producing monoclonal RF precede lymphoma development in patients’ SS, often by several years. Additionally, B cell clonal expansions may frequently be detected in the salivary glands of SS patients by PCR-based techniques [66]. It is important to note that different clones can be found in different tissues at different times indicating that some clones are yet to evolve into malignant lymphoma [67].

Analysis of the IgVH genes used by sialadenitis-associated clones from different SS patients revealed a limited repertoire of VH genes such as VH1-69, or VH3-7, enhanced mutational frequencies and similar CDR3 sequences, all of which suggest that a unique antigen may be driving the B cell proliferation. These findings also indicate that these B cell clones represent a highly selected B cell population with enhanced ability to bind to the same or identical antigen. The negative selection against replacement mutations within CDR3 also suggests that the immunoglobulin antigen receptor plays an important role in the selection and expansion of B cell clones [68]. It has been reported that VH1-69 and VH3-7 rheumatoid factors have characteristic CDR3s that are approximately 12 to 14 amino acids long, preferentially use JH4 and JH3, respectively, and bear conserved amino acid sequences in VD and DJ junctions, similar to that found in sialadenitis-associated B cell clones. VH gene analysis therefore suggests that several B cell clones express immunoglobulins with RF activity [68].

Expression of antibodies with RF activity is a frequent characteristic of MALT B cell lymphomas. Analysis of Immunoglobulin VH-CDR3 sequence of a panel of B NHLs revealed the expression of B cell antigen receptors with strong CDR3 homology to canonical V1-69 and V3-7 RFs in salivary MALT lymphomas expressed [69,70]. Of note, Bende et al. showed that 41% of salivary gland lymphomas expressed B cell antigen receptors with strong CDR3 homology to RF, compared to only 18% of gastric MALT lymphomas and none of the pulmonary MALT lymphomas that were included in the study [69].

IgG autoantibodies produced locally on the salivary glands of patients with SS may form ICs which in turn chronically stimulate B cells expressing RF B cell receptor (BCR) and Toll like receptor (TLR)-7 [71,72,73,74]. Inefficient immunosurveillance of these RF B cells may lead to clonal expansion and lymphomatous evolution (for further information see Section 3.5 and Section 3.7.1).

Hence, we can safely say that SS is characterized by aberrant clonal expansion of RF B cells. The nature of this monoclonal B cell expansion whether small, large, localized, disseminated, established or fluctuating, may imply a different risk for lymphoma development.

#### 3.3.3. FcRL4 Expressing B Cells

FcRL4 is an immunoregulatory receptor, selectively expressed by B cells with a marginal zone phenotype localized in the subepithelial regions and within the epithelium of tonsils and Peyer patches (marginal zone equivalents) [75,76] Neoplastic B-cells-associated MALT lymphomas, especially those involved in LELs, have been shown to express FcRL4 [76]. Haacke et al. demonstrated that FcRL4^+^ B cells are proliferating in LELs and that FcRL4 expression is associated with parotid MALT lymphoma developing in the setting of pSS [38].

The FcRL4^+^ B cell subset found in SGs of pSS patients is known to present enhanced TLR signaling and dampened response to BCR signaling [40,77]. Zheng et al. demonstrated that lymphocytes located in parotid LELs of pSS patients present increased TLR9 expression compared to controls [78]. Epithelial cells, both through apoptosis, that provides ligands for TLR activation, and production of cytokines can drive the sustained activation and proliferation of FcRL4^+^ B cells [79,80].

Upregulation of genes associated with lymphomagenesis in FcRL4^+^ B cells has been recently shown, leading to the hypothesis that neoplastic MALT B-cells may arise from glandular FcRL4^+^ intraductal B cells [42]. Based on IGHV gene analysis Visser et al. demonstrated the presence of B cell clones not only restricted to the ducts, but also seen in the periductal areas, indicating a possible translocation of clonal between the two compartments [81]. FcRL4^+^ B cells within the SGs of pSS patients can express activation-induced- deaminase (AID) [38,42,82]. Given the increased proliferative activity, the potency of AID expression and their association with LEL, one could attribute to intraductal B cells characteristics capable of leading them to neoplastic evolution. Viser et al. also demonstrated that most of the B cell clones found in their study did not express BCRs with homology for stereotypic RF sequences, a finding indicative of clonal expansion independent of a BCR with RF activity [81]. Thus they hypothesize that activated B cells migrate into the striated ducts in the SGs of patients with SS, irrespective of BCR specificity, while transformation to pathogenic RF expressing B cells is the result of lymphoma driver mutations acquisition, under the effect of the stimulatory environment of the striated duct [81,83].

FcRL4^+^ B cells are found in higher numbers in parotid glands of SS patients compared to labial glands, a finding compatible with the fact that SS associated lymphoma develops more frequently in the parotids of SS patients [38].

### 3.4. Ectopic Germinal Center Formation

Although salivary gland histology reveals a predominance of activated T cells in early lesions of SS patients, B cells predominate in severe histologic lesions. This strong B cell infiltration is not only a morphologic phenomenon, but it is also progressively associated with the formation of ectopic GCs. An array of cytokines, adhesion molecules and chemokines regulates the complex process of ectopic GC formation in SS [84]. Several studies emphasize the importance of ectopic GC formation in the pathogenesis of SS associated lymphoma.

Physiologically B cell selection takes place in the GCs of lymphoid tissues. GCs are specialized microstructures of the secondary lymphoid tissues. In GCs B cells undergo somatic hypermutation of the variable Ig heavy and light chain genes, leading to the emergence of B cell clones that bind antigen with high affinity. Regulation of the processes taking place in GCs is critical to ensure self-tolerance by limiting the production of auto-reactive B cell clones [85].

Ectopic lymphoid structures can develop at sites of chronic inflammation in peripheral, non-lymphoid organs, leading to the expression of phenotypic features characteristic of secondary lymphoid organs. These structures are characterized by the segregation of T cells and B cells in discrete areas, more specifically B cell follicles surrounded by T cell rich areas, in association with the development of follicular dendritic cell (FDC) networks. In SS, studies have estimated the prevalence of ectopic lymphoid structures to 30–40% [86].

It has been shown that these GC-like structures within the salivary glands of SS patients are not merely a histological finding but exhibit a functional activity. Autoantibody-producing cells were detected at higher frequency and numbers in SS patients with GCs compared to patients lacking such structures [87]. Stott et al. showed that locally Ag driven somatic Ig variable heavy and light gene hypermutation in salivary gland GC of SS patients allows affinity maturation of GC B cells [59]. Ectopic GCs express the molecular machinery necessary to support local autoantibody production and B cell expansion. AID is the enzyme responsible for class switch recombination and somatic hypermutation of the Ig genes. AID expression is exclusively seen in B cells undergoing class switch recombination (CSR) and somatic hypermutation (SHM), rendering its detection sufficient to address the question of whether ectopic germinal center B cells activate their molecular machinery responsible for hypermutation of Ig genes [88]. Bombardieri et al. demonstrated that AID expression is an invariable finding within the FDC networks, while the enzyme is not detectable in SGs in the absence of ectopic GC-like structures, proving that FDCs are crucial for the Ag- driven B cell proliferation within the SS salivary glands [89]. Though we should consider the need for standardization in the assessment of the germinal center in the SG of SS patients, since the FDC networks are indeed a prerequisite for GC formation, their identification is not necessarily indicative of GC presence.

Though GCs are the major sites of clonal B cell expansion and SHM of rearranged Ig genes, we should consider that SHM may take place in B cell compartments other than the germinal center [90,91]. In rodent secondary lymphoid organs, CSR and low-levels of SHM have been demonstrated to take place at extrafollicular sites [92]. It can also be expressed by extrafollicular activated B cells of normal human lymphoid tissues (tonsils, lymph nodes and spleen) and interfollicular large B cells outside the ectopic GC in the T-cell rich areas of periductal aggregates containing FDC networks of SS-MSG and SS parotids [89,93,94]. Moreover, as described in Section 3.3.2 FcR4L+ intraductal B cells can express activation-induced- deaminase (AID) [38,42,82].

Carubi et al. failed to prove an association between ectopic GC like structures and lymphoma, though the study demonstrated a high prevalence of ectopic germinal like structures (56%) and a lower prevalence of lymphoma (2%, only 2 patients, who both had ectopic GC like structures) [95]. A prospective Swedish study by Theander et al. demonstrated that 14% of SS patients with GC-like structures developed lymphoma, in contrast to 0.8% of patients with no GC like structures (*p* = 0.001), with a median onset of seven years after the initial diagnostic salivary gland biopsy. GC-like structures were present in 25% of the patients at diagnosis. Six of the seven patients that developed lymphoma had GC-like structures at diagnosis [23]. Yet in this study, GC like structure presence was evaluated in MSGs, while in five out of seven lymphoma cases it developed in organs other than the SGs [23]. Opposing evidence, though, is demonstrated by the study of Haacke et al., where the presence of GCs in labial gland biopsies from patients that subsequently developed parotid MALT lymphoma was not identified as a predictive factor for lymphoma development, though only anti-SSA positive patients and only patients with parotid gland lymphoma were included in the study, not representing the diversity of SS patients [96]. Johnsen et al. also failed to demonstrate an association between ectopic GC formation and lymphomagenesis, though their study was not designed to evaluate the relative risk of lymphoma occurrence in association with GC like structures, included a smaller number of patients and used a different method for GC estimation [97]. More recently, Sene et al. demonstrated that the presence of ectopic GC-like structures in MSGis an independent risk factor of lymphoma occurrence in SS patients, with a 7.8-fold increased risk, while SG lymphoma was only reported in 2 out of 8 lymphoma patients [28]. Given the contradictory results of the above-mentioned studies, we should highlight the need for standardization for the uniform evaluation of ectopic germinal centers, as well as the need for evaluation of ectopic GCs in other tissues infiltrated by lymphoma in SS patients.

Parameters considered as predictors of lymphoma development, namely anti-Ro/SSA and anti-La/SSB antibodies, hyperglobulinemia, salivary gland swelling, higher focus score and extra-glandular manifestations have been associated with the presence of ectopic germinal-center like structures [87,95,98,99].

Interestingly, Szodoray et al. identified biomarkers that can discriminate SS patients based on the presence of ectopic GCs. The biomarkers having the strongest discriminatory capacity for GC presence were CCL11, IFN-γ and BAFF, a finding consistent with the fact that ectopic germinal center formation is regulated by the action of various cytokines, chemokines and adhesion molecules [100].

### 3.5. The Role of BAFF

After the establishment of lymphoid infiltration in the SGs of SS patients, CD4^+^ T cells and DCs produce cytokines that promote B-cell survival and proliferation, including BAFF [33,101]. BAFF, a member of the tumor necrosis factor (TNF) ligand family and an essential factor of B cell activation and proliferation, is a potential player in SS-related B cell deregulation [102]. BAFF has no effect on B-cell tolerance in the bone marrow, but does act in the periphery, after the T1 immature B-cell stage and is essential for the survival of T2 cells and down-stream B subsets [103]. BAFF acts regulates B cell selection, with increased competition among auto- and alloreactive B cells for BAFF leading to elimination of autoreactive B cells and decreased competition for BAFF, due to increased levels of circulating BAFF, resulting in relaxation of BAFF selection and escape of autoreactive naive B cells [103]. BAFF was found to be increased in salivary glands of SS patients [33,44,104,105],while BAFF receptor (BAFF-R) expression is decreased in SS B-lymphocytes, possibly due to BAFF overexpression, and its decrease correlates with disease activity [106]. Moreover, BAFF levels have also been correlated with autoantibody titers in SS patients [107].

The production of high levels of BAFF leads to the engagement of the numerous BAFF receptors expressed on salivary B cells. As a result, the peripheral check point against autoreactivity fails with subsequent emergence of autoreactive B cells in the GCs and MZ equivalents. Consequently, the ectopic lymphoid structures in SS offer a microenvironment suitable for autoreactive B cells’ abnormal activation and expansion via a T cell dependent pathway. Although the degree of T-cell independent immune response in SS remains unclear, excess BAFF may be central in the progression of autoimmune process.

Increased BAFF concentrations may contribute to lymphomagenesis through persistent B cell activation [108]. Higher BAFF levels are found in primary SS patients with lymphoma or pre-lymphomatous manifestations compared to those without [109]. Serum BAFF levels have been correlated with higher ESSDAI and clonal B cell expansion in salivary glands [110]. Gottenberg et al. demonstrated that BAFF levels remain high in pSS that have developed lymphoma, even years after lymphoma treatment and remission, suggesting a genetic origin of such a persistent increase in BAFF levels. Studies of polymorphisms in BAFF loci demonstrated an association with increased BAFF levels [109]. Gotteneberg et al. showed no association between the -871 T/C promoter polymorphism in BAFF gene and pSS [111]. Nezos et al. demonstrated that distinct BAFF haplotypes confer increased risk or protection for lymphomagenesis, implicating the host’s genetic background in pSS related lymphomagenesis [112]. Mutations of the BAFF-R gene (namely His159Tyr) showed increased prevalence in patients with SS, particularly those that developed MALT lymphoma. Of interest, more than two-thirds of SS patients that developed MALT lymphoma with an age at SS diagnosis between 3rd and 4th decade carried this mutation [113]. In the setting of BAFF-RHis159Tyr mutation, activation of the alternate NF-kB pathway may contribute to lymphomagenesis [113,114].

### 3.6. The Role of Cytokines

The SS phenotype includes distinct molecular subtypes defined by characteristicIFN signatures [115]. Data show that both type I and type II IFN signatures are upregulated in peripheral blood and minor salivary glands of SS patients, while IFNγ/IFNα ration in MSG tissues could discriminate a subgroup a patients at risk for lymphoma development [116]. IFNα induces the tumor suppressor gene p53, the extrinsic apoptotic molecule TRAIL and the Ro52 autoantigen, a SS autoantigen that negatively regulates the anti-apoptotic protein Bcl-2. Nezos et al. demonstrated that IFNα transcript levels were remarkably reduced in the MSG tissues of SS patients that developed lymphoma compared to SS patients without lymphoma [116]. IFNα levels were correlated with mRNA expression of pro-apoptotic molecules TRAIL, p53 and Ro52, leading to the conclusion that IFNα suppression can lead to the survival of malignant B cell populations.

Other cytokines have also been implicated in chronic B cell activation and lymphoma development is SS. Levels of Fms-like tyrosine kinase 3 ligand (Flt-3L), a cytokine implicated in B cell ontogenesis and proliferation in hematologic malignancies, have been found to be elevated in patients with SS and correlated with abnormal B cell distribution [117].

Serum levels of two other cytokines implicated in GC formation, CXCL13 and CCL11, have been correlated with SS disease activity and lymphoma development [118].

### 3.7. Nuclear Factor Kappa B (NF-κB) Pathway

#### 3.7.1. Tumor Necrosis Factor-Alpha Induced Protein 3 (TNFAIP3) and Control of Immune Activation

Chronic stimulation of B cells by ICs in the microenvironment of SS SGs requires functional checkpoints of autoimmune B cell activation to prevent lymphomatous escape. The *TNFAIP3* gene encodes A20 protein. A20 protein, an enzyme with ubiquitination activity, is rapidly induced after NF-κB activation and is involved in the negative feedback regulation of NF-κB signaling in response to specific pro-inflammatory stimuli, thus controlling both apoptosis and inflammation [119]. TNFAIP3 has been found to be down-regulated in salivary gland epithelial cells from patients with SS compared to controls and the cells with down-regulated TNFAIP3 expression exhibited enhanced NF-κΒ activities [120]. Somatic and germline mutations of *TNFAIP3* have been correlated functional abnormalities of the protein with a frequency as high as 77% in SS patients that developed MALT lymphoma [121]. A coding *TNFAIP3* variant (rs2230926) has been correlated to lymphoma development in patients with SS of French and UK origin [122]. In a large Greek cohort of SS patients, increased prevalence of the rs2230926 mutant variant was detected in both non-lymphoma SS and lymphoma-SS patients compared to controls, with the rs2230926 mutant variant being detected in approximately one fifth of SS-lymphoma patients with disease onset ≤40 years, leading to the conclusion that NF-κB pathway dysregulation results in increased lymphoma susceptibility especially in patients with early disease onset [123]. In the setting of continuous B cell stimulation by autoimmunity, germline abnormalities of genes leading to inefficient control of the NF-κΒ activation enhance the risk of lymphoma [122]. Johnsen et al. demonstrated weaker TNFAIP3 immunoreactivity in minor salivary glands of pSS patients with lymphoma than in those without lymphoma [124].

#### 3.7.2. MYD88

MyD88 is an adaptor protein leading to NF-κB activation, through TLR, IL-1R and IL-18 signaling. The presence of MyD88 L265P mutation has been implicated in the pathogenesis of Waldenström macroglobulinemia (WM) and other hematological malignancies [125]. Given that WM has been associated with several immune mediated disorders, including SS [126]. Voulgarelis et al. studied the possible implication of MyD88 L265P mutation in the pathogenesis of SS-related lymphoproliferation. MyD88 L265P somatic mutation in SS patients with and without lymphoma was not detected, indicating that the mechanisms of lymphomagenesis in SS are different from those of WM and other hematological malignancies [127].

### 3.8. Oncogenic Events

#### 3.8.1. BCL2 Dysregulation

*BCL2* codes for an antiapoptotic protein initially described in follicular lymphomas, where the chromosomal translocation t(14;18) leads to BCL2 overexpression and promotes B cell survival. Pisa et al. detected the translocation t(14;18) was in five of seven SS-associated lymphomas, while among patients with SS not complicated by lymphoma no bcl-2 translocations were detected in 50 consecutive SG biopsies [128]. The frequency of the classical t(11;18)(q21;q21) translocation was found to be lower in SS patients with extra-gastrointestinal compared to those with gastric MALT lymphoma [129]. Genome wide DNA profiling of MALT lymphomas identified gain of function in cytoband 18q, which includes *BCL2*, a finding that correlated with gene expression [130]. Reksten et al. demonstrated an association between the intronic single nucleotide polymorphism (SNP) rs4940574 in *BCL2* and GC status in patients with SS, suggesting a possible link with lymphoma development in these patients [131].

#### 3.8.2. Tumor Suppressor Genes

Mutations of the tumor suppressor gene *p53* are possibly associated with lymphomagenesis in SS [132] (Tapinos, 1999).

#### 3.8.3. Other Cytogenetic Abnormalities

The presence of trisomies 18, 3, and 12 has been demonstrated in SS associated MALT lymphomas of the SGs. Of note, the transition from benign lymphoepithelial lesion, where no chromosomal aberrancies are found, to MALT lymphoma is characterized by an increase in frequency and number of numerical aberrations and DNA. Peri-tetraploid DNA non-diploidy might be characteristic for high grade MALT lymphoma of the salivary gland [133].

### 3.9. Other Mechanisms Possibly Implicated in SS Lymphomagenesis

#### 3.9.1. Inflammasome

Recently the role of the inflammasome has been outlined in the pathogenesis of SS and SS lymphomagenesis. P2X7 receptor (P2X7R) promotes inflammatory responses via the NLPR3 inflammasome. P2X7 was found upregulated in SG biopsies of patients with SS, stimulating IL-18 production. This upregulation was restricted to epithelial cells and correlated with the presence of GC like structures and increased risk of MALT lymphoma development [134]. More recently Vakrakou et al. demonstrated that SS patients at high risk for lymphoma development, as well as SS patients that have developed lymphoma, displayed a unique NLRP3 inflammasome gene signature in peripheral blood mononuclear cells and increased levels of IL-18 [135].

#### 3.9.2. Methylation

The role of epigenetics is SS lymphomagenesis have recently been studied. SNPs of the methylene-tetrahydrofolate reductase (MTHFR) gene, an enzyme essential in DNA synthesis and methylation, have been associated with susceptibility to non-MALT NHL development in SS patients [136].

Mavragani et al. investigated the possible implication of altered DNA methylation in the inappropriate expression of LINE-1 (L1) retroelements in pSS. Reduced levels of L1 promoter methylation along with increased DNA methyltransferase (DNMT)3B, DNMT1 and MeCp2, but reduced LSH levels were detected in SS-low risk patients compared to both SS-lymphoma and sicca controls. The SS-lymphoma group was also characterized by a profound decrease of MeCP2 and DNMT3B compared to sicca controls [137]. These data support the role of altered methylation mechanisms in the pathogenesis of SS and SS related lymphomagenesis.

#### 3.9.3. MicroRNAs (miR)

miR200b-5p levels in minor salivary glands have been shown to be a strong predictive biomarker for lymphoma development in SS. Its expression levels in MSGs were downregulated long before the clinical onset of lymphoma, implicating micro-RNA deregulation as another mechanism implicated in SS lymphoma pathogenesis [138].

## 4. Summarized Model of Lymphomagenesis in SS

**Figure 1:**
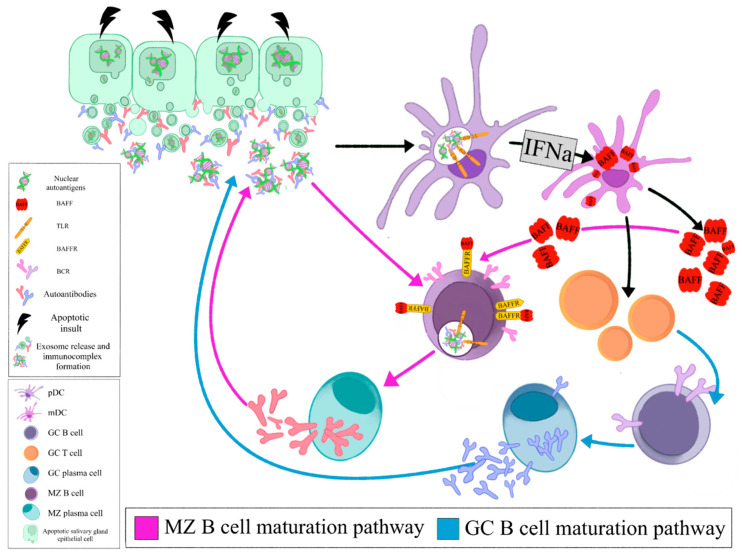
Opsonized epithelial apoptotic particles and ICs containing nuclear autoantigens bind to plasmacytoid DCs (pDCs) and RF B cells via TLRs and Fc receptors, respectively. TLR activation in pDCs leads to IFNa release that subsequently stimulates myeloid DCs (mDCs) to produce BAFF. The same apoptotic particles and ICs also bind to RF B cells via TLR and BCR, respectively. TLR activation in B cells results in enhanced BCR mediated signaling and upregulation of BAFF-R. BAFF, secreted by mDCs, further upregulates TLR expression, favors B cell survival, promotes immunoglobulin class- switching and plasma cell differentiation (MZ B cell maturation pathway). The activated mDCs also act as antigen presenting cells to T cells that help B cell responses (GC B cell maturation pathway). (pDC: plasmacytoid dendritic cell, mDC: myeloid dendritic cell, GC: germinal center, MZ: marginal zone, BAFF: B cell activating factor, TLR: Toll like receptor, BAFFR: BAFF receptor, BCR: B cell receptor, IFNa: interferon a).

The local auto- antigen expression by the epithelium of the LEL in SGs of SS patients drives the emergence of autoreactive B-cell clones bearing an RF reacting BCR. Opsonized epithelial apoptotic particles and ICs containing nuclear autoantigens bind to plasmacytoid DCs (pDCs) and RF B cells via TLRs and Fc receptors, respectively. TLR activation in pDCs leads to IFNa release that subsequently stimulates myeloid DCs (mDCs) to produce BAFF. The same apoptotic particles and ICs also bind to RF B cells via TLR and BCR, respectively. TLR activation in B cells results in enhanced BCR mediated signaling and upregulation of BAFF-R. BAFF, secreted by mDCs, further upregulates TLR expression, favors B cell survival, promotes immunoglobulin class-switching and plasma cell differentiation (Figure 1, Z B cell maturation pathway). The activated mDCs also act as antigen presenting cells to T cells that help B cell responses (Figure 1, GC B cell maturation pathway). Therefore, the combined signals of BCR, TLR, BAFF and IFNa drive the propagated production of autoantibodies and lead to expansion of RF B cells (Figure 1) [139].

Selection of these RF-expressing B cells offers them a proliferative advantage, leading to their malignant transformation and eventually MALT lymphoma development. Autoreactive MZ B cells have been shown to be negatively selected in healthy individuals. RF-expressing, autoreactive MZ like B cells in SS bypass the peripheral checkpoint against autoreactivity and proceed to proliferation and differentiation through T cell independent pathways [140].

## 5. Conclusions

**Figure 2:**
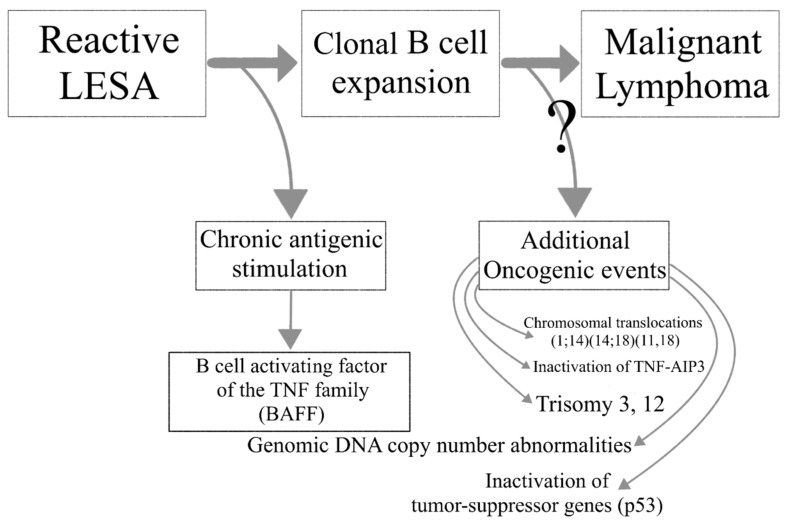
The transition from lymphoepithelial sialadenitis to malignant lymphoma.

Lymphoma development in the setting of SS is associated with increased overall disease mortality. A multistep process leads to the transition of reactive LESA to lymphomagenesis. Chronic antigenic stimulation leads to abnormal B cell activation in the SG glands of SS patients and the emergence of autoreactive B cell clones. Loss of immune control, ectopic GC formation and oncogenic events further drive the malignant transformation to lymphoma (Figure 2).

## Figures and Tables

**Table 1:** Predictors of lymphoma development in SS.

Predictive Factors	References
Clinical	
Permanent parotid enlargement	[3,9,10,11]
Splenomegaly	[13]
Lymphadenopathy	[3,9,10,11,12,13,21]
Palpable purpura	[8,9,14]
Peripheral neuropathy	[15]
Biological	
Cryoglobulinemia	[8,13,16,17,18,21,22]
Lymphopenia	[14,16,17,19]
Low complement levels	[8,9,11,13,14,15,17,19,21]
Monoclonal component in serum or urine	[11,15,16,17,20]
GC-like structures in SG biopsy, FS	[23,24,28]

GC: Germinal Center, SG: Salivary Gland, FS: Focus Score.
